# A Case of Trephining

**Published:** 1863-11

**Authors:** T. Wilkins

**Affiliations:** Greenville, Bond Co., Illinois


### A CASE OF TREPHINING.

By T. WILKINS, M. D., of Greenville, Bond Co., Ills.

Samuel T. Bryant, 27 years of age, and belonging to the service of the United States, was temporarily remaining in this county. On the 18th of June he was wounded by the breech-pin of an old musket, which was blown out when he fired it and struck him in the forehead, near the median line. On one side, the skull was depressed near the half of an inch. The mind was undisturbed, and when I saw him, five or six hours after the accident, he had recovered from the shock. This being his situation, and the fatality so great after the operation of Trephining, I concluded not to interfere in this way without urgent necessity. One eye was so injured from the explosion and the powder, that it was lost beyond redemption. After being cleansed, this and the wound of skull were dressed with simple warm water dressing. Morphine, in moderate doses, was given to quiet.

June 19th and 20th.—Rests well. Continue treatment. The case continued much the same till the 29th, when he became feverish and delirious, when blood was taken from his arm till an impression was made on the artery, and the following was given: ℞. Morphine Sulph., gr. ij; Hydr. Chloride, gr. vj. Mix and divide into vj equal doses. S. One every three hours.

June 30th.—No particular alteration in the case. Continue Morphine, and determine to operate as soon as possible, but could not do so until next day, July 1st, when sufficient integument was turned back to enable me to place a small Trephine on a sound portion of the skull. After removing a small crescent shaped piece of bone, I was enabled to remove the fractured portions. Of these there were not less than a dozen, broken in all shapes, and the inner ones pressing on the dura mater which was not broken. After the operation, there was a breach 1½ inches long by 1¼ wide extending to the orbit of one eye and including a part of the median line. But little blood was lost.

The brain was throbbing with life, and after cleansing a compress and bandage only was applied. From loss of integument it was impossible to close the wound as advised by authors. Indeed, with due deference to their opinion, I think the wound ought to be left open. There is far more danger from pent up pus causing inflammation of the brain than there is of the contact of the air.

Before the operation brandy was given, after which he was placed under the influence of chloroform, and, to digress a little, allow me to say, I never give chloroform without first giving an alcoholic stimulant, and no untoward symptoms ever follow in my practice. For the time being, it keeps the heart and arteries in motion. One of your correspondents gives it as his opinion that death (after Anæsthesia) is caused from heart clot. If this be true, it explains the advantage of alcoholic stimulants in such cases.

To return, the delirium soon began to leave my patient after the operation, and in about two days he was again rational. Three large abscesses formed on him, one on the neck, another over the deltoid muscle, and the third over the supra scapular muscle. Several weeks after the operation he took erysipelas, which extended from his face and neck over a large part of his body. This all left him. He then took a catarrh, but has since become able to go to his brother, Dr. Bryant, who practices in Sycamore, Illinois. During the whole of his treatment (after operation) he took large doses of morphine whenever there were symptoms of irritation. His safety, so far, I think, to a great extent may be attributed to this article. Tonics and other articles were used as indicated.

